# Supplementary material for: Active site specificity profiling datasets of matrix metalloproteinases (MMPs) 1, 2, 3, 7, 8, 9, 12, 13 and 14
Source: Data Brief. 2016 Feb 22;7:299–310. doi: 10.1016/j.dib.2016.02.036 (PMC4777984; doi:10.1016/j.dib.2016.02.036)
Supplement: Supplementary file 10 — Supplementary material [file mmc10.zip › WebPICS_hMMP2_G_1%/P2.html]

 

PICS results


|  |  |
| --- | --- |
| **P2\_Q**  18 in 246 sites   7.3 %    effects > 10 perc. pnts.  (vice-versa in brackets)  P2prime\_I: 24.7 (12.7)   P2prime\_Q: 11.0 (14.1) |  |
